# Resuscitative endovascular balloon occlusion of the aorta in civilian pre-hospital care: a systematic review of the literature

**DOI:** 10.1186/s40001-022-00836-3

**Published:** 2022-10-17

**Authors:** Yaset Caicedo, Linda M. Gallego, Hugo JC. Clavijo, Natalia Padilla-Londoño, Cindy-Natalia Gallego, Isabella Caicedo-Holguín, Mónica Guzmán-Rodríguez, Juan J. Meléndez-Lugo, Alberto F. García, Alexander E. Salcedo, Michael W. Parra, Fernando Rodríguez-Holguín, Carlos A. Ordoñez

**Affiliations:** 1 Centro de Investigaciones Clínicas (CIC), Fundación Valle del Lili, Cra. 98 No. 18 - 49, Valle del Cauca, Cali, Colombia; 2 Facultad de Medicina, Universidad Icesi, Cl. 18 No. 122 - 135, Valle del Cauca, Cali, Colombia; 3 Instituto de Ciencias Biomédicas, Facultad de Medicina, Universidad de Chile, Av. Libertador Bernardo O’Higgins 1058, Santiago de Chile, Región Metropolitana Chile; 4 Department of Surgery, Caja Costarricense del Seguro Social, Av. 2nda - 4rta Cl. 5nta - 7tima, San José, Costa Rica; 5 Division of Trauma and Acute Care Surgery, Department of Surgery, Fundación Valle del Lili, Cra. 98 No. 18 - 49, Valle del Cauca, 760032 Cali, Colombia; 6 Division of Trauma and Acute Care Surgery, Department of Surgery, Universidad del Valle, Cl. 13 # 100 - 00, Valle del Cauca, Cali, Colombia; 7 Division of Trauma and Acute Care Surgery, Department of Surgery, Hospital Universitario del Valle, Cl. 5 # 36 - 08, Valle del Cauca, Cali, Colombia; 8 Department of Trauma Critical Care, Broward General Level I Trauma Center, 1600 S Andrews Ave, Fort Lauderdale, FL USA

**Keywords:** REBOA, Pre-hospital, Civilian, Return of spontaneous circulation, Hemorrhagic shock, Cardiac arrest

## Abstract

**Background:**

Resuscitative endovascular balloon occlusion of the aorta (REBOA) is a damage control tool with a potential role in the hemodynamic resuscitation of severely ill patients in the civilian pre-hospital setting. REBOA ensures blood flow to vital organs by early proximal control of the source of bleeding. However, there is no consensus on the use of REBOA in the pre-hospital setting. This article aims to perform a systematic review of the literature about the feasibility, survival, indications, complications, and potential candidates for civilian pre-hospital REBOA.

**Methods:**

A literature search was conducted using Medline, EMBASE, LILACS and Web of Science databases. Primary outcome variables included overall survival and feasibility. Secondary outcome variables included complications and potential candidates for endovascular occlusion.

**Results:**

The search identified 8 articles. Five studies described the use of REBOA in pre-hospital settings, reporting a total of 47 patients in whom the procedure was attempted. Pre-hospital REBOA was feasible in 68–100% of trauma patients and 100% of non-traumatic patients with cardiac arrest. Survival rates and complications varied widely. Pre-hospital REBOA requires a coordinated and integrated emergency health care system with a well-trained and equipped team. The remaining three studies performed a retrospective analysis identifying 784 potential REBOA candidates.

**Conclusions:**

Pre-hospital REBOA could be a feasible intervention for a significant portion of severely ill patients in the civilian setting. However, the evidence is limited. The impact of pre-hospital REBOA should be assessed in future studies.

**Supplementary Information:**

The online version contains supplementary material available at 10.1186/s40001-022-00836-3.

## Background

The resuscitative endovascular balloon occlusion of the aorta (REBOA) is a useful tool in the hemodynamic resuscitation of severely ill traumatic and non-traumatic patients [1, 2]. REBOA allows hemorrhage control and maintains perfusion towards vital organs. This endovascular tool has been used as a bridge to definitive management [3–5]. Therefore, the potential benefit of the implementation of a REBOA as part of pre-hospital resuscitation management has been suggested [6, 7]. The role of pre-hospital REBOA in severely injured civilian trauma patients has already been revisited by the Committee on Trauma of the American College of Surgeons. They acknowledge that most of the United States Emergency Medical Services (US EMS) systems are not prepared for this intervention, and it should occur only as part of a clinical trial with specific recommendations. However, other countries with advanced prehospital systems are performing this intervention [8]. This article aims to perform a systematic review of the literature about the feasibility, survival, indications, complications and potential candidates for civilian pre-hospital REBOA.

## Methods

This systematic review was performed using the Preferred Reporting Items for Systematic reviews and Meta-Analyses (PRISMA) guidelines [9]. A predetermined selection protocol including potential objectives, inclusion/exclusion criteria, search methods, and data analysis techniques was registered in the PROSPERO, ID: 197542 (https://www.crd.york.ac.uk/prospero/display_record.php?RecordID=197542) (Additional file 1: Text—Table S1).

### Eligibility criteria

The eligibility criteria were patients of any age who required REBOA placement before emergency room admission regardless of the underlying cause (traumatic or non-traumatic). Also, studies that retrospectively evaluated the potential candidates for pre-hospital REBOA were included. Indications for REBOA were defined by each study. Studies conducted on military trauma were excluded.

### Information sources and search strategy

A comprehensive literature search was conducted using MEDLINE (PubMed), Embase, Web of Science, and LILACS (Literatura Latinoamericana y del Caribe en Ciencias de la Salud) databases. The search terms were: “Reboa OR Aortic balloon tamponade OR Resuscitative endovascular balloon occlusion AND Pre-hospital management OR Pre-hospital care OR Out of hospital OR Ambulance”. The reference list of the identified studies was also searched. No restrictions were made based on language, publication date, or publication status. The final search was performed on December 26th, 2021 (Additional file 1: Text—Table S2).

### Selection and data collection process

All studies were identified by two review authors (YC; NP) who independently searched databases, using a standardized extraction form (Microsoft Excel—Microsoft Corp, Redmond, WA, USA). Two blinded reviewers (NP; HC) selected the possible eligible studies according to titles and abstracts. Any disagreement between reviewers was resolved by a third author (YC). Two reviewers in a blinded standardized fashion verified the inclusion and exclusion criteria in the selected articles. The following data were extracted and recorded: author, year of publication, title, objective, type of study, inclusion criteria, methods, primary outcomes, secondary outcomes, other results, and conclusions. Four authors were assigned for this task (LG; CG; HC; NP). A fifth author (YC) resolved any disagreements.

### Data items

Primary outcomes were survival, feasibility (defined as the number of patients in whom prehospital REBOA was successfully placed among the total of patients in whom the procedure was attempted) and compliance to eligibility (defined as the proportion of eligible patients in whom the procedure was attempted). Secondary outcomes were complications, potential pre-hospital REBOA candidates, return of spontaneous circulation (ROSC) and cardiopulmonary resuscitation (CPR) requirements.

### Study bias assessment

The modified Methodological Index for Non-Randomized Studies (MINORS) was used to assess the methodological quality of all studies [10]. Two independent authors (NP; IC) evaluated the study quality and any discrepancies were resolved by a third author (YC).

### Synthesis methods

A great heterogeneity was observed among the studies in terms of criteria for REBOA placement, studied population, objectives, and methods. Therefore, it was not possible to perform a meta-analysis. Studies were classified in trauma and non-trauma patients and analyzed based on methodological features and results. We performed a qualitative analysis of the survival, feasibility, and potential use of REBOA in the civilian pre-hospital setting.

## Results

### Study selection

**Fig. 1:**
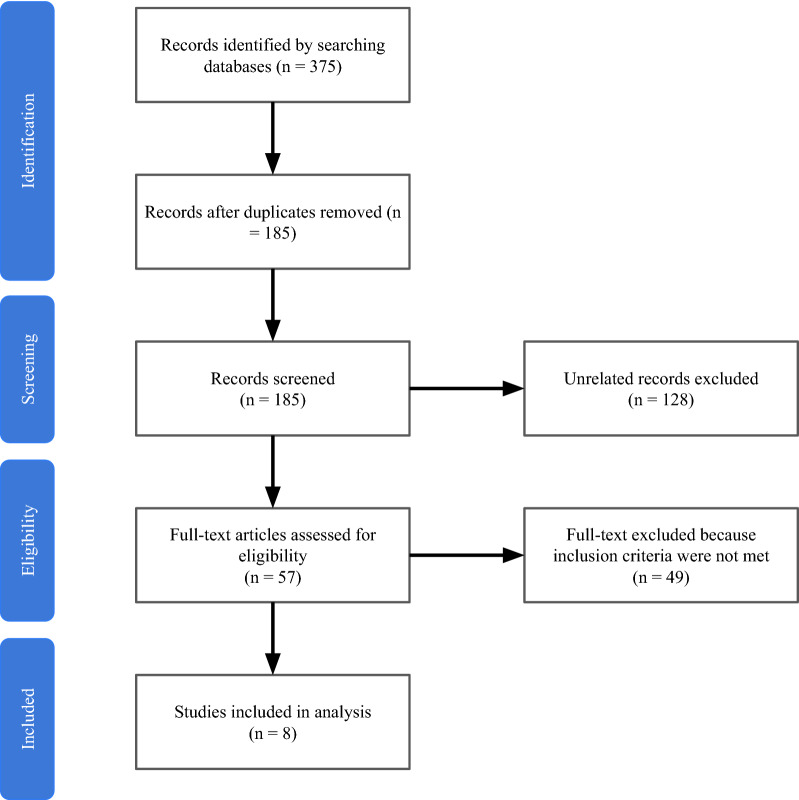
PRISMA flowchart diagram showing the selection process of the studies. *N* = 8 number of articles

A total of 375 articles were identified through electronic search, of which 190 were duplicates. One hundred and twenty-eight studies were excluded based on irrelevant titles and/or abstracts. The remaining 57 studies were evaluated in full-text detail and 49 were excluded. Finally, 8 studies (3 case series, 2 retrospective cohorts, and 3 cross-sectional studies) were included in the analysis (Fig. 1) [6, 11–17]. These studies were published between 2016 and 2021 and conducted in Norway, France, Italy, and the United Kingdom and the United States (Tables 1, 2, 3).

**Table 1:** Characteristics of civilian prehospital teams with experience in the use of REBOA

Author-year	Prehospital team	Team skills	Endovascular training	Times reported
Sadek 2016 [11] (London—United Kingdom)	London’s Air Ambulance (LAA) based at the Royal London Hospital: The experienced physician–paramedic team provides a 24-h dedicated trauma service to the 10 million inhabitants of London, attending approximately 1800 patients per year. The hospital is a Major Trauma Center and has approximately 3500 full trauma team activations per year All REBOA procedures were performed by physicians with multi-specialty backgrounds including Emergency Medicine, Anesthesia, and Intensive Care Medicine	The pre-hospital team is trained in advanced prehospital interventions such as rapid sequence of anesthesia, blood transfusion and resuscitative thoracotomy. The team is able to activate in-hospital major hemorrhage protocols A multidisciplinary working group to investigate the pre-hospital REBOA potential role was created. The group consisted of clinicians from pre-hospital care, emergency medicine, trauma surgery, interventional radiology, anesthesia, and intensive care medicine	A protocol for pre-hospital REBOA was produced along with a structured training, education, and governance program. In addition, the Pre-hospital, and Emergency Department Endovascular Resuscitation (PEER) Course was created to disseminate knowledge within the wider pre-hospital and in-hospital team Training included scripted scenarios used in high-fidelity, training “moulages” to test the trainees’ leadership, decision-making, teamwork, and procedural competence [19]	Injury to arrival on scene: 34 min Dispatch to procedure start: NR Dispatch to occlusion, min: NR Procedure time, min: NR Occlusion time, min: NR Dispatch to ROSC, min: NR
Lendrum 2019 [12] (London—United Kingdom)				Injury to arrival on scene, median (IQR): 21 (18–26) min Dispatch to procedure start, min: NR Dispatch to occlusion, min: NR Procedure time, min: NR Occlusion time, median (IQR): 80 min (75–115) min Dispatch to ROSC, min: NR
Brede 2019 [15] (Trondheim—Norway)	Trondheim´s Helicopter Emergency Medical Service (HEMS) based at St. Olavs Hospital: The physician-leaded HEMS has a catchment population of about 700.000 and usually transfer patients with OHCA to this tertiary university hospital. The service dispose both a helicopter and a rapid response car All physicians are board certified qualified anesthesiologist with prehospital work experience from 4 to 18 years. The paramedics have from 11 to 34 years work experience in the service. Eight physicians and 5 paramedics participated, and all completed a structured training program before entering the study	All anesthesiologists are skilled in establishing central vascular lines using the Seldinger technique and ultrasound. The team was capable of assuring an optimal advanced cardiac life support (ACLS) resuscitation, using a chest compression machine and performing endotracheal intubation. They also measured invasive arterial BP via the left radial or brachial artery at 1-min intervals in the 2021 cohort They created a safety monitoring group specifically focused on correct catheter placement and the quality of advanced resuscitation, following a 3-step safety assurance system	The training program included theoretical education, training on a special designed simulation mannequin, training during elective angiography procedures, and high-fidelity simulation Performance was evaluated with a global rating scale and all participants had to perform above a predefined score to complete the training program. Details of the training program have been reported [18]	Dispatch to arrival on scene, min: NR Dispatch to procedure start: NR Dispatch to occlusion, mean (range) 45.6 (34–57) min Procedure time, mean (range) 11.7 (8–16) min Occlusion time, mean 9.5 (3–19) min* Dispatch to ROSC, mean: 53.3 (37–58) min *Occlusion times are only indicated for patients with ROSC
Brede 2021 [17] (Trondheim—Norway)				Dispatch to arrival on scene, median (IQR):29 (10–38) min Dispatch to procedure start: NR Dispatch to occlusion, median (IQR): 50 (39–72) min Procedure time, min: NR Occlusion time, min: NR Dispatch to ROSC, mean (range): 53.5 (50–57) min
Gamberini 2021 [16] (Bologna—Italy)	Bologna´s Helicopter Emergency Medical Service (HEMS) based at Maggiore Carlo Alberto Pizzardi Hospital: This hospital is a level 1 Trauma and stroke center with 927 beds. It also includes the Emergency Medical Services Dispatch center and the local HEMS base covering a 2.5 million inhabitants’ area. They receive an average of 180 OHCA patients per year from both EMS and HEMS For procedures managed by the HEMS crews, a UCI Intensivist performs REBOA assisted by one of the two HEMS nurses while the second nurse ensures that quality CPR is delivered by the crews of the ground vehicles dispatched together with HEMS	The prehospital team is capable of assuring ACLS and performing maneuvers such as finger thoracostomy, pericardiocentesis and eFAST. If necessary, chest compression device and portable ventilators are available All the attending intensivists have a significant experience in ultrasound-guided arterial cannulation because of the trauma management background	The REBOA technique was acquired by the trauma ICU intensivists in 2015 and the same group of 17 physicians covers a 24 h/7d shift in the local HEMS Each member of the team directly performed or collaborated to at least two REBOA procedures before conducting the technique independently. Mandatory simulation-based retraining is performed every 6 months	Dispatch to arrival on scene, median (IQR): 12.5 (6–16.5) min Dispatch to procedure start, median (IQR): 26.5 (24.5–46.5) min Dispatch to occlusion, median (IQR): 38 (34.5–48.5) min Procedure time, median (IQR): 9 (9–10.75) min Occlusion time, min: NR * Dispatch to ROSC, min: NR* *No available data due to not achieved sustained ROSC in the Prehospital group. Median time of occlusion was 32 min for all the patients (ED and HEMS)

*REBOA* resuscitative balloon occlusion of the aorta, *PEER* pre-hospital and emergency department endovascular resuscitation, *NR* not reported, *ROSC* recuperation of spontaneous circulation, *IQR* interquartile range, *HEMS* Helicopter Emergency Medical Service, *OHCA* out-of-hospital cardiac arrest, *ACLS* advanced cardiovascular life support, *EMS* Emergency medical services, *CPR* cardiopulmonary resuscitation, *eFAST* extended focused assessment with sonography in trauma, *ED* emergency department

**Table 2:** Experience of REBOA in the pre-hospital setting

Study	Type of study	Participants	Interventions	Outcomes	Conclusions
Sadek 2016 [11] (United Kingdom)	Case report	*N* = 1 A 32-yo severely injured patient, with exsanguinating hemorrhage secondary to multiple pelvic fractures	Zone III REBOA, insertion under ultrasound guidance Introducer Sheath 8 Fr and Balloon Catheter 7 Fr (14 mm)	Primary outcomes: Feasibility: REBOA was successfully performed Survival: The patient survived to hospital discharge (52 days) without neurological impairment Compliance to eligibility: N/A Secondary outcomes: There were no complications and CPR was not required ROSC: N/A	Prehospital REBOA is possible and may contribute to manage severe NCTH
Lendrum 2018 [12] (United Kingdom)	Case series	*N* = 21 Patients with NCPH and hemodynamic instability: - 19 from traumatic origin - 2 from non-traumatic origin	Zone III REBOA, insertion under ultrasound guidance Introducer Sheath 7 Fr and Balloon Catheter 6 Fr (13 mm) A pre-alert call was made to the receiving major trauma center	Primary outcomes: Feasibility: 15 (71%) patients out of 21 attempts underwent a successful REBOA procedure - Traumatic: 13/19 (68%) - Non-traumatic: 2 out of 2 Survival: 60% (9/15) survived to hospital discharge: - Traumatic: 8/13 (62%) - Non-traumatic: 1 out of 2 Compliance to eligibility: Not reported Secondary outcomes: - CPR was not required - ROSC: 1 non-traumatic patient in cardiac arrest achieved ROSC following REBOA - Early arterial thrombosis following REBOA was present in 77% (10/13) of trauma patients - Other complications were amputation, SFA cannulation, inadvertent zone II placement, and iatrogenic dissection of the CFA to distal aorta	Prehospital REBOA is a feasible resuscitation strategy for patients with NCTH in a physician-led pre-hospital care system Pre-hospital Zone III REBOA may reduce the risk of pre-hospital hypovolemic cardiac arrest and early death due to exsanguination Distal arterial thrombus formation is common and should be expected and actively managed
Brede 2019[15] (Norway)	Prospective cohort study	N = 15 Patients with non-traumatic OHCA, aged 18 to 75 years and in which CPR was initiated in less than 10 min after onset of arrest	Zone I REBOA. insertion under ultrasound guidance The Introducer Sheath size was not reported, and the Balloon Catheter was 7 Fr (20 mm) All patients received CPR using a chest compression machine to standardize cardiac massage	Primary outcomes: Feasibility: Prehospital REBOA was successfully performed in the 10 attempted procedures (100%) - 8 in the first attempt - 2 in the second Survival: 30% (3/10) survived to hospital admission and 1 to the 30-day follow-up Compliance to eligibility: Prehospital REBOA was attempted in 10 of 15 (66%) eligible patients Secondary outcomes: All patients received CPR and there were no complications ROSC: 6/10 patients (60%) achieved ROSC	This study shows the feasibility and safety of prehospital REBOA as an adjunct treatment to non-traumatic OHCA, without interfering with the ACLS quality
Brede 2021 [17] (Norway)	Prospective cohort study	*N* = 17 Patients with non-traumatic OHCA, aged 18 to 75 years and in which bystander CPR was initiated in less than 10 min after onset of arrest	Zone I REBOA. insertion under ultrasound guidance The Introducer Sheath size was not reported, and the Balloon Catheter was 7 Fr (20 mm) All patients were endotracheally intubated, manually ventilated and received mechanical chest compressions	Primary outcomes: Feasibility: Prehospital REBOA was successfully performed at first cannulation attempt in the 7 attempted procedures (100%). However, 2 patients were excluded from the study due to extra-arterial placement of the peripheral arterial line Survival: 20% (1/5) survived to hospital admission but not to the 30-day follow-up Compliance to eligibility: Prehospital REBOA was attempted in 7 of 17 (41%) eligible patients Secondary outcomes: All patients received CPR and no complications were reported ROSC: 2/5 patients (40%) achieved ROSC	This study suggests that REBOA as an adjunct treatment during resuscitation may significantly increase the peripheral arterial blood pressures and it is likely that this indicates improved central aortic blood pressure
Gamberini 2021 [16] (Italy)	Case series	*N* = 20 Patients with refractory OHCA (defined as lack of ROSC after 15 min of CPR, in the absence of hypothermia) who were not eligible for ECPR REBOA was placed in the ER (*n* = 12) or pre-hospital setting (*n* = 8). The later were: - 4 trauma patients - 4 non-trauma patients	Zone I REBOA. insertion under ultrasound guidance Initially Introducer Sheath 8 Fr and Balloon Catheter 8 Fr (30 mm). After June 2019, Introducer Sheath 7 Fr and Balloon Catheter—Fr (32 mm) Non-trauma patients underwent cardiothoracic and abdominal ultrasound prior to REBOA Trauma patients underwent bilateral thoracostomy, eFAST and pericardiocentesis (if necessary) prior to REBOA	Primary outcomes: Feasibility: Prehospital REBOA was successfully performed in the 8 attempted procedures (100%) Survival: There were no survivors Compliance to eligibility: Not reported Secondary outcomes: All patients received CPR and no complications were reported ROSC: 3/8 patients (38%) achieved ROSC - Traumatic: 1/4 (25%) - Non-traumatic: 2/4 (50%)	This series of mixed cases suggests that a transient ROSC can be achieved, despite suffering from refractory cardiac arrests with long low flow times. However, survival may be influenced by the long times to ROSC and late application of the technique during CPR

*ACLS* advanced cardiovascular life support, *CFA* common femoral artery, *CPR* cardiopulmonary resuscitation, *ECPR* extracorporeal cardiopulmonary resuscitation, *ER* emergency room, *eFAST* extended focused assessment with sonography in trauma, *NCTH* non-compressible torso hemorrhage, *NCPH* non-compressible pelvic hemorrhage, *OHCA* out-of-hospital cardiac arrest, *REBOA* resuscitative balloon occlusion of the aorta, *ROSC* recuperation of spontaneous circulation, *SFA* superficial femoral artery

**Table 3:** Potential candidates for pre-hospital REBOA

Study	Methods	Participants	Outcomes	Conclusions
Trauma studies				
Thabouillot 2018 [13] (France)	Retrospective cross-sectional study Analysis of all the trauma patients registered in the Paris Fire Brigade database January 1st, 2014, to December 31st, 2014	*N* = 1159 Eligible candidates: Adults with suspected abdominal, pelvic, or junctional bleeding, uncontrolled hemorrhagic shock (SBP < 90 mmHg) and cardiac arrest or pressor amine requirement ≥ 5 mg/h	Main outcome: 3.2% (37/1159) were considered candidates for pre-hospital REBOA Other outcomes: - Median ISS 29 (25–34) - The global out-of-hospital death rate with conventional management was 83.8% (31/37) - The mechanisms of injury were falls (59.5%), car crash (21.6%), train collisions (10.8%), and stab/gunshot wounds (8.1%)	This is the first study to propose the eligibility criteria for pre-hospital REBOA, which includes high dose amine use, emphasizing that REBOA should be used as a last resource and only when benefits outweigh risks
Henry 2019 [14] (United States)	Retrospective cohort study Review of full autopsies of patients with traumatic cardiac arrest who arrived at a Level I Trauma Center in Los Angeles January 2014 to March 2018	*N* = 198 Eligible candidates: Those who, based on autopsy findings, suffered abdominal organ injuries and/or pelvic fractures as a source of NCTH, with no severe head injuries (AIS ≥ 3)	Main outcome: 13.6% (27/198) were considered candidates for pre-hospital REBOA Other outcomes: -Median ISS 22 (17–29) - Most of these patients had severe injuries (AIS ≥ 3): 85.2% (23/27) had abdominal solid organ injuries and 65.4% (17/27) had pelvic fractures	This study concludes that there is a potential role for REBOA in prehospital settings and that some clinical variables could identify the patients that most likely will benefit from this lifesaving intervention
Non-trauma study				
Brede 2020 [6] (Norway)	Retrospective cohort study Analysis of the patients with OHCA captured by the Norwegian Cardiac Arrest Registry January 1st, 2016, to December 31st, 2018	*N* = 8339 Eligible candidates: Those aged 18 to 75 years, with witnessed cardiac arrest, suspected non-traumatic etiology, ambulance response time < 15 min and CPR duration > 30 min “Potentially eligible” candidates: Same indications as above but CPR duration between 15–30 min	Main outcome: 8.6% (720/8339) were considered candidates for pre-hospital REBOA Other outcomes: - 6.3% (528/8339) were considered “potentially eligible” candidates for pre-hospital REBOA - The cohort overall survival at 30-day follow-up was 14%, with good neurological outcomes in 83% of the cases - Presumed non-traumatic cardiac arrest causes were cardiac in 1543 (78.6%), respiratory in 276 (14.1%), overdose/intoxication in 69 (3.5%) and strangulation in 76 (3.9%)	This study suggests that there is sufficient patient population in Norway to study REBOA as an adjunct treatment in non-traumatic OHCA

*AIS* Abbreviated Injury Scale, *CPR* cardiopulmonary resuscitation, *GCS* Glasgow Coma Score, *ISS* injury severity score, *NCTH* non-compressible torso hemorrhage, *OHCA* out-of-hospital cardiac arrest, *REBOA* resuscitative balloon occlusion of the aorta, *SBP* systolic blood pressure, *SpO2* oxygen saturation

### Risk of bias

The studies that analyzed the pre-hospital REBOA outcomes have a MINORS score of 8 to 13 points and the studies related to the potential pre-hospital REBOA candidates have a score of 5 to 6 points. Therefore, the included studies had a high to moderate risk of bias (Additional file 1: Text—Tables S3, S4).

### Individual study results

#### Emergency team and technical conditions for REBOA

Three civilian emergency teams from London (the United Kingdom), Trondheim (Norway), and Bologna (Italy) described their experience with prehospital REBOA placement [11, 12, 15–17]. All the emergency teams have rapid response systems with air medical service based at level-I hospitals and their team members include physicians with expertise in REBOA. The prehospital teams underwent specific REBOA training, with education strategies supported by simulation [13, 18, 19]. Additionally, mandatory simulation-based retraining is performed every 6 months by the Italian emergency team [16]. The prehospital care teams were capable of performing advanced resuscitation maneuvers such as rapid sequence induction of anesthesia, early hemostatic resuscitation, resuscitative thoracotomy, pericardiocentesis, and/or finger thoracostomy [16, 18, 19]. Most of the patients who required CPR were attended with mechanical chest compression devices (described by three studies); moreover, the Italian team also employed portable ventilators [15–17]. The constitution of the advanced emergency teams, personnel skills, and training characteristics are detailed in Table 1.

The prehospital REBOA indications were heterogeneous between researcher groups. REBOA was indicated in trauma patients with hemodynamic instability due to non-compressible pelvic hemorrhage (NCPH) and refractory out-of-hospital cardiac arrest (OHCA) [11, 12, 16]. The indication in non-trauma patients was refractory cardiac arrest or CPR maneuvers initiated in less than 10 min after the onset of arrest. Small-gauge introducers (7–8 Fr) were used in all studies. The balloon catheter was inflated in the aortic zone I for patients with traumatic or non-traumatic OHCA and in the aortic zone III for trauma patients with NCPH. Studies reported that all procedures were performed under ultrasound guidance (Table 2).

#### Primary outcomes

Among the five studies that described pre-hospital REBOA placement, two included non-trauma patients [15, 17], other two included trauma and non-trauma patients [12, 16], and the fifth one was a case report of a trauma patient [11]. A broad variability was found in the feasibility, survival and compliance to eligibility reported by the studies (Table 2). The majority of trauma cases were reported by the British emergency team. In 2016, Sadek et al. published the first case report of a catastrophic pelvic hemorrhage patient managed with REBOA who survived until hospital discharge without neurological impairment [11]. 2 years later, the same emergency British team attempted the procedure in 21 patients with non-compressible pelvic hemorrhage (NCPH). REBOA placement was successful in 13 trauma patients (13/19) with a survival rate at hospital discharge of 62% (8/13 patients) [12]. Eligible patients in whom the procedure was not attempted were not reported. With respect to non-trauma patients, the Norwegians published two studies including patients in CPR initiated within 10 min of OHCA. In 2019, Brede et al. conducted a successful intervention in all 10 patients in whom the procedure was attempted (10/10), with a survival rate of 30% (3/10) at hospital admission and 10% (1/10) at 30-day follow-up. The procedure was not performed in 5 eligible cases [15]. An extension of this study was performed 2 years later, they reported 41% of compliance to eligibility (7/17) and a feasibility of 100% (7/7) with just 1 patient admitted to the hospital, who died before the 30-day follow-up [17]. Gamberini et al. attempted the prehospital procedure in 8 patients with refractory OHCA from both traumatic and non-traumatic etiology. REBOA was achieved in 4 trauma and 4 non-trauma patients, but none of them survived [16]. Eligible patients in whom the procedure was not attempted were not reported. A detailed information of each study could be found in Table 2.

#### Relevant times and complications

Regarding the response and procedural times, not all studies provide complete information. However, according to four studies, arrival times at the scene ranged from 12.5 to 34 min [11, 12, 16, 17]. Gamberini et al. also indicate that median procedure start time from emergency dispatch was 26.5 min (IQR 24.5–46.5) [16]. Two studies informed the time from dispatch to balloon inflation with a median of 38 and 50 min [16, 17], and Brede (2019) a mean of 45.6 min (34–57) [15]. The procedural times were reported with a mean of 11.7 min (8–16) and median of 9 min (IQR 9–10.75), by Brede (2019) and Gamberini (2021), respectively [15, 16]. Concerning the balloon occlusion times, there was notorious variation, Lendrum reported a median of 80 min (IQR 75–115) and Brede (2019) a mean of 9.5 min (3–19) [12, 15]. Specified times by each researcher group are listed in Table 1. Gamberini and Brede noted that the REBOA procedure did not add unnecessary time on scene as an adjunct to standard advanced life support, furthermore Brede observed no delay in the transport to hospital [15–17].

Complications were not informed by the Italian and Norwegian studies [15–17]. Otherwise, the British team stated that the first reported case did not suffer from complications or sequelae until hospital discharge (52 days after injury) [11]. However, in the subsequent case series from 2018 they found frequent complications following REBOA [12]. These complications were predominantly early arterial thrombosis, observed in 10 of 13 trauma patients (77%) who required embolectomy/thrombectomy and in which 6 were directly related to a traumatic vascular injury. Other less common complications included inadvertent superficial femoral artery (SFA) cannulation requiring patch angioplasty, inadvertent zone II placement causing renal infarcts and iatrogenic dissection of the common femoral artery (CFA) to distal aorta [12]. Additionally, 4 patients from this British case series required lower limb amputation (3 unilateral and 1 bilateral). There were no significant differences in the amputation rate comparing to unsuccessful REBOA group (31% [4/13] vs 50% [3/6], *p* = 0.617). Brede et al. specified that there were no adverse events associated with the intervention or negative influence on the quality of standard advanced life support [15]; rather they demonstrated increases in peripheral arterial pressure [17]. Lendrum also observed significant improvement in systolic blood pressure after the intervention [12].

#### Potential candidates

Three retrospective studies aimed to define the potential patients who could have benefited from pre-hospital REBOA in trauma and non-trauma population [6, 13, 14]. These three retrospective studies found that 3.2% (37/1159) of all trauma patients, 13.6% (27/198) of traumatic cardiac arrests and 8.6% (720/8339) of ambulance-treated cardiac arrests could benefit from prehospital REBOA (9, 22, 23). Thabouillot and Henry et al. determined the potential REBOA candidates including abdominopelvic trauma patients with uncontrolled hemorrhagic shock [13, 14].Henry et al. proposed the following criteria for pre-hospital REBOA: Glasgow Coma Scale ≥ 9 (*p* = 0.012, OR 3.20), Systolic Blood Pressure < 90 mmHg (*p* = 0.04, OR 4.31), and/or Oxygen Saturation > 90% (*p* = 0.03, OR 7.28)[14].

For the non-traumatic population, Brede et al. followed a cohort of OHCA patients over a 3-year period, they found 720 (8.6%) candidates and 528 (6.3%) “potentially eligible” candidates, acknowledging that “potentially eligible” might become “eligible” if the response and procedure times were shorter. Presumed non-traumatic cardiac arrest etiologies were cardiac in 1543 (78.6%), respiratory in 276 (14.1%), overdose/intoxication in 69 (3.5%) and strangulation in 76 (3.9%) [6]. Each studied population and the eligibility criteria for the potential REBOA candidates are outlined in Table 3.

## Discussion

To our knowledge, this review is the first to summarize the current evidence of REBOA in the civilian pre-hospital setting. We found limited evidence with a low-to-moderate quality and wide variability in REBOA indications and outcomes. REBOA is a low-frequency procedure with high dexterity requirement. Thus, the evidence remains without high-quality prospective controlled studies. Therefore, we propose the development of multi-institutional studies with international collaboration, to enlarge the sample and achieve the homogenization of protocols, indications and outcome measures.

### Emergency team training

There are several training courses about REBOA implementation [20, 21]. However, the lack of validity evidence for the assessment tools difficult the guiding on how to ensure competence [20]. All emergency teams from the included studies, used different courses and protocols (two designed their own). The three prehospital teams described a simulation-based training [16, 18, 19]. A recent systematic review found a favorable effect on procedural competence with simulation-based training regardless of the type of simulator and the outcome measures used [20]. However, they recognize that existing data on REBOA training are scarce and low quality, therefore evidence-based guidelines are needed on how to train REBOA and on how to ensure competence. Furthermore, literature on REBOA training does not include any assessment of long-term follow-up [22]. A decline in proficiency level must be expected with time. Hatchimonji et al. reported that clinical performance deteriorate 6 months post-course without clinical practice [22], which suggest that REBOA refresher training should be considered at 6-month intervals.

### Primary performer of REBOA insertion

All REBOA procedures included in this systematic review were performed by physicians with multi-specialty backgrounds including emergency medicine, anesthesia, and intensive care medicine. Clinicians with an appropriate skill set and specific REBOA training, can successfully accomplish this pre-hospital intervention. Available literature informed that in almost 10% of in-hospital REBOA insertions, the primary performer is not the trauma/acute care surgery attending (remaining 91%). Clinicians vary from trauma/acute care surgery fellow, surgery resident, vascular surgery attending, interventional radiology attending or emergency medicine attending [21]**.** Moreover, emergency physicians (and fellows under supervision) have shown they can effectively place REBOA, without diminishing the survival rates observed in case series of trauma surgeons [23]. The effectiveness of a short training paves the way for the use of REBOA by emergency physicians in austere conditions [24].

### Technical issues of the pre-hospital REBOA insertion

There are several challenges regarding the implementation of REBOA in the pre-hospital setting. Factors to consider are the prehospital personnel skills, type of prehospital care delivered, and transport mode [25]. The first challenge in the use of REBOA is to achieve vascular access [26, 27].

All procedures were performed under ultrasound guidance by attending physicians. In addition, the prehospital advanced emergency teams should be well-equipped and integrated with the emergency health care system. We found that all teams had air transport, with most crews using two teams to avoid delays or interference with standard management. In patients under CPR, chest compression machines and monitoring teams were available for ensuring quality. Therefore, the use of REBOA requires multidisciplinary health personnel with training and advanced equipment. This can be a disadvantage in low to middle-income countries, and limit the applicability of these techniques [28–30].

The reviewed studies acknowledged that even with a strict protocol, there are several factors in out-of-hospital settings that might interfere [11, 12, 15, 17]. Factors such as constricted space, scarce lightening, cold weather, limited personnel, environmental hazards, or insecure road conditions were identified. These could explain why REBOA was not placed in 5 eligible patients of Brede´s first cohort and in 10 of the second cohort [15, 17]. Previous reports also mentioned that lighting and visualization proved to be appreciable impediments in the context of a simulated military readiness exercise [31].

### Times on scene

Prehospital times informed by the included studies report relatively short response times with balloon inflation within the “Golden Hour” (less than 50 min). Also, it appears that less time is needed to decide the intervention comparing to in-hospital attempts, without scene or to hospital transport delays. However, incomplete information and lack of uniformity in definitions prevent us from reaching a conclusion. Systematic review on the influence of prehospital times in trauma patients stated that literature endorse the “stay-and-treat” approach, rather than the “scoop and run” [25]. This is supported by the finding of increased odds of survival with longer time spent on the scene, which they accredit to the comprehensive care that is delivered prehospitally. In the same study, the arrival to hospital within the “Golden Hour” fails to decrease mortality in 2 out of 3 studies that report on this matter, suggesting that prehospital advanced interventions could be more beneficial to make the most of this precious hour. In consequence, this could imply that for the future, the emphasis should not be on getting a patient to the hospital as fast as possible, but making sure the patients receive proper prehospital care first.

On the other hand, balloon occlusion times were rarely reported and highly variable. The Norwegian team informed a mean of 9.5 min (3–19), while the British a median of 80 min (IQR 75–115) [12, 15]. This variation could be explained by differences in protocols, team training, and patient indications. Especially for trauma patients, it is important that this variable is reported, as prehospital REBOA could prolong aortic occlusion, perhaps increasing the risk of ischemia–reperfusion injury [32]. Duration of aortic occlusion is directly related to the degree of physiological consequences of distal ischemia and reperfusion. To overcome this limitation, regional permissive hypotension through partial occlusion has been used. Partial REBOA allows prolonged occlusion, preserving distal blood flow and reducing ischemia or organ injury [33]. Controlled clinical trials are necessary to enlighten whether or not prehospital REBOA lengthens occlusion times and partial REBOA could be the solution.

### Pre-hospital REBOA in trauma patients

In the civilian trauma population, pre-hospital REBOA is feasible in 68 to 100% of the cases, with a survival rate to hospital discharge ranging from 0 to 62%. This variability could be explained by the technical issues previously discussed.

Indications of pre-hospital REBOA in trauma patients are relatively clear and mirror in-hospital indications [34]. The indications described in the studies included were: NCPH patients with hemodynamic instability and/or refractory OHCA. It has been observed that REBOA can safely control non-compressible torso hemorrhage in both blunt and penetrating trauma patients with lower risk-adjusted odds of mortality in penetrating trauma [35, 36]. The refractory OHCA group has lower survival rates, most likely due to the precarious hemodynamic condition of these patients. Aortic occlusion before a cardiac arrest could increase the probability of survival. A critical threshold of 70 mm Hg of systolic blood pressure has been proposed for an ideal cutoff for the aortic occlusion [37, 38]. CFA access should be obtained in all patients with a high risk of hemodynamic collapse [26, 27, 39]. A recent review of literature suggests that prehospital REBOA is likely futile in patients with an asystolic arrest from exsanguination. However, REBOA can be considered in patients with a profound hypovolemic shock to prevent cardiac arrest as part of the pre-hospital Endovascular Trauma Management (EVTM) [3, 40, 41, 44]. We recommend that future studies should evaluate early aortic occlusion.

Only Lendrum reported complications, including early arterial thrombosis, in which 6 out of 10 cases were not directly related to groin access [12]. Perhaps, correlated with small-gauge introducers (7–8 Fr) employed in all studies. Small vascular-sheaths are related to lower overall rate of vascular complications [22, 27, 34]. Similar to Lendrum experience, evidence suggests that lower limb amputation directly related to vascular puncture for REBOA insertion is uncommon [34, 42]. However, complications can arise in arterial access, balloon positioning, deflation, or other stages of REBOA placing. Thus, more solid, prospective evidence of the complications at each stage is needed.

Evidence suggests that 3.2% of all trauma patients and 13.6% of traumatic cardiac arrests could potentially benefit from a pre-hospital REBOA. Nevertheless, this data should be carefully interpreted since the physiologic parameter cutoff points were arbitrarily decided. Henry et al. proposed three clinical variables (GCS ≥ 9, SBP < 90 mmHg, and SaO2 > 90%) with a 100% positive predictive value to identify REBOA candidates [14]. These parameters should be considered and assessed in future studies to establish homogeneous indications for civilian pre-hospital REBOA in trauma.

### Pre-hospital REBOA in non-trauma patients

In the non-trauma population, pre-hospital REBOA was primarily used in OHCA. The Italian team performed the procedure in patients with refractory cardiac arrest (lack of ROSC after 15 min of CPR) who were not eligible for ECPR. In the Norwegian cohorts the indication was CPR maneuvers initiated in less than 10 min after the onset of arrest. Once again, in the Italian study the selection criteria used favored the enrollment of patients with an extremely low expected probability of survival, compared to the other studies. This could explain the difference in survival rates.

Our review found that in this population REBOA was feasible (100% of cases) and safe without impact on the Advanced Life Support quality. However, a brief pause in chest compressions is necessary to achieve a vascular access and a second emergency crew is required. In addition, ultrasound verification of correct catheter placement during CPR is challenging and not always reliable due to chest/abdomen movement and gastric/intestinal air from bag-mask ventilation [43]. Severe vasoconstriction due to high cumulative doses of adrenaline may difficult arterial access reducing feasibility rates. These additional technical aspects should be considered in future studies aiming to perform this intervention.

Efficacy outcomes such as ROSC (40–60%) and survival to hospital admission (0–30%) had a wide variability and are inconclusive. The current rates of ROSC and survival to hospital discharge following OHCA are lower than 25% and 10%, respectively. These outcomes remain essentially unchanged since 2012 [44–46]. These have been attributed to the inability of traditional interventions to sufficiently increase coronary perfusion pressure (> 15 mm Hg) even under optimal conditions [47, 48]. A growing information of preclinical and clinical evidence suggests that REBOA may increase the coronary and cerebral arteries blood flow, perfusion pressure, and/or rates of ROSC [47, 49–51].

However, higher mortality and a longer time to arterial access is expected and several clinical trials are required to evaluate the potential benefit and safety of this intervention. A multicenter, randomized, parallel group, clinical trial (REBOARREST) is underway expecting to determine the efficacy of pre-hospital REBOA as an adjunct treatment in non-traumatic OHCA [52].

## Limitations

We acknowledge that this review has several limitations, including the low-to-moderate quality of the studies and the potential selection and information bias. There was wide variability in the clinical indications and outcome measures for REBOA, limiting our ability to develop conclusions. Likewise, some outcomes were not reported such as additional interventions, hemostatic resuscitation, time to definitive hemorrhage control, in-hospital treatment, among others. To overcome this frequently encountered limitation, a consensus on a Core Outcome Set for REBOA clinical trials was developed [53]. This should help enable higher-quality evidence, leading to more significant conclusions. Finally, these results cannot be applied to low/middle-income countries because the available information comes from high-income countries with physician-lead emergency teams, properly trained, well equipped, with rapid response, and air transportation supported by level I hospitals.

## Conclusion

Evidence related to REBOA in the civilian pre-hospital setting is low-quality. Pre-hospital REBOA could be a feasible intervention for a select proportion of traumatic and non-traumatic patients. However, its implementation requires a coordinated and integrated emergency health care system with well-trained and equipped teams. It is paramount to achieve consensus regarding indications for REBOA and evaluate the benefit of earlier aortic occlusion. Further studies are required for a better understanding of the impact of this prehospital intervention on balloon occlusion times and associated complications. Clinical trials are needed to assess the efficacy and safety of pre-hospital REBOA.

## Supplementary Information

**Additional file 1**: **Table S1**. PRISMA Guidelines. **Table S2**. Boolean algorithms for the review use of REBOA in prehospital setting. **Table S3**. Modified MINORS Score. **Table S4**. Evaluation of risk of bias according to modified MINOR score.

## Data Availability

The datasets generated and/or analyzed during the current study are available in the PROSPERO, ID: 197542 (https://www.crd.york.ac.uk/prospero/display_record.php?RecordID=197542).

## Abbreviations

US EMS: United States Emergency Medical Services
CFA: Common femoral artery
CPR: Cardiopulmonary resuscitation
EVTM: Endovascular trauma management
GCS: Glasgow Coma Scale
IQR: Interquartile range
LILACS: Literatura Latinoamericana y del Caribe en Ciencias de la Salud
MINORS: Methodological index for non-randomized studies
NCPH: Non-compressible pelvic hemorrhage
OHCA: Out-of-hospital cardiac arrest
PRISMA: Preferred Reporting Items for Systematic reviews and Meta-Analyses
REBOA: Resuscitative endovascular balloon occlusion of the aorta
ROSC: Return of spontaneous circulation
SBP: Systolic blood pressure
SFA: Superficial femoral artery
